# Multifactorial intervention for diabetes control among older users of insulin

**DOI:** 10.11606/S1518-8787.2018052000144

**Published:** 2018-05-07

**Authors:** Rafael Vaz Machry, Henrique Umpierre Pedroso, Luthiele Silva Vasconcellos, Rafaela Ramos Nunes, Cibelle de Abreu Evaldt, Eduardo Bardou Yunes, Ticiana da Costa Rodrigues

**Affiliations:** I Universidade Federal do Rio Grande do Sul. Faculdade de Medicina. Departamento de Medicina Interna. Porto Alegre, RS, Brasil; II Universidade Federal do Rio Grande do Sul. Programa de Pós-Graduação em Endocrinologia. Porto Alegre, RS, Brasil; III Universidade Federal de Santa Maria. Centro de Ciências da Saúde. Departamento de Clínica Médica. Santa Maria, RS, Brasil

**Keywords:** Aged, Diabetes Mellitus, prevention & control, Glycemic Index, drug effects, Hypoglycemic Agents, Insulin, administration & dosage, Blood Glucose Self-Monitoring, Clinical Trial

## Abstract

**OBJECTIVE:**

To evaluate if the closer follow-up with the supply of insulin pens and the measurement of capillary blood glucose improve the management of older patients with type 2 diabetes without adequate glycemic control despite extensive therapy.

**METHODS:**

This is a prospective, non-randomized, quasi-experimental study. We have included 45 patients over 60 years old, from both sexes, with glycated hemoglobin (HbA1c) > 8.5% using oral hypoglycemic agents and insulin. The intervention consisted of monthly medical visits, with the provision of insulin pens and strips for blood glucose measurement. All patients received insulin pen, refills of Neutral Protamine Hagedorn and regular insulin, needles for the pen, blood glucose meter, and capillary blood glucose tests (three tests/day). Treatment was adjusted with the same endocrinologist monthly for six months. Glycated hemoglobin was measured at baseline and 12 and 24 weeks after intervention.

**RESULTS:**

Glycated hemoglobin at baseline was 10.34% (SE = 0.22%) and 8.54% (SE = 0.24%, p < 0.001) and 8.09% (SE = 0.21%, p < 0.001) at 12 and 24 weeks after intervention, respectively, with a significant reduction from baseline.

**CONCLUSIONS:**

More frequent medical visits, with treatment inputs including the use of insulin pens and self-monitoring, have improved glycemic control (reduction of 2.25% in HbA1C, on average, at 24 weeks of follow-up). Our data support a change in the management and medical behavior of older patients with chronically decompensated diabetes.

## INTRODUCTION

Patients with type 2 diabetes (T2D) may need to use insulin as part of treatment to achieve adequate glycemic control. In Brazil, according to the *Vigilância de fatores de risco e proteção para doenças crônicas por Inquérito Telefônico* (VIGITEL) 2015, which is a population-based study, the number of patients with diabetes is increasing, and 24.4% of the population over 65 years of age reported having diabetes in 2014^1^. Nevertheless, the care of these patients is hindered by the lack of access to current and new medical monitoring and therapeutic technologies. A large study with T2D patients has shown that only 26% of them had good glycemic control in the public healthcare system^2^.

It is essential to ensure adherence to treatment in order to achieve satisfactory glycemic control. Approximately 34.6% of patients with diabetes report that they miss applying insulin at least once a month^3^. The number of days without insulin or with non-adherence is higher among patients with obstacles to insulin use (they had more difficulty in applying the injection and were not satisfied with the flexibility of insulin injections or presence of hypoglycemia)^3^. On the other hand, using insulin pens to improve adherence is associated with reduced healthcare costs^4^. Patients using pens find it easier to use the insulin and fewer report pain on application^5^.

Pawaskar et al.^6^ have studied patients using a syringe compared to insulin pens and they have observed that syringe users had higher health care costs, although the direct costs of the insulin delivery method are higher for pen users. However, the response to the replacement of syringes by pens in older patients with chronic decompensated diabetes in clinical trials is not known.

Older patients tend to have higher rates of adherence than younger ones^7^; however, they also need attention, and there are few studies on this population. We have evaluated older patients with diabetes treated in the Brazilian public healthcare system who already use high doses of insulin and have no adequate glycemic control for long periods. We have developed this study including patients aged 60 years or older with chronically uncontrolled diabetes who are already monitored at a tertiary hospital. All patients were reassigned to monthly medical follow-up with frequent adjustments in treatment, use of insulin pens, and daily self-monitoring of blood glucose (SMBG).

## METHODS

This is a quasi-experimental study to examine a multiple intervention to improve glycemic control, including the replacement of syringes by insulin pens in older patients with T2D without adequate glycemic control in a Brazilian tertiary hospital. The Research Ethics Committee of the *Hospital de Clínicas de Porto Alegre* has approved the study protocol. All participating patients provided written informed consent. Funding was provided by *Conselho Nacional de Pesquisa* (CNPq) - Brazil and *Fundo de Incentivo à Pesquisa* (FIPE) of *Hospital de Clínicas de Porto Alegre*.

We invited patients who had been previously scheduled for consecutive medical visits from the Diabetes Section - Endocrinology Division of *Hospital de Clínicas de Porto Alegre*. Patients were included between June and December 2014.

We included patients with chronically uncontrolled T2D, 60 years or older, already using Neutral Protamine Hagedorn [NPH] insulin, either in association with regular insulin or not, with syringes in addition to at least one oral antihyperglycemic agent. We considered patients as having chronically inadequate control if glycated hemoglobin (HbA1c) were equal or greater than 8.5%, collected less than three months before. There were no limits for higher values of HbA1c. We did not include persons who self-declared as unable to self-administer insulin or who had a glomerular filtration rate lower than 30 ml/min/1.73 m^2^ by the MDRD equation.

All participants received insulin pens, needles for the pens, NPH and regular insulin, as well a blood glucose meter and lancets. Patients were instructed on the use of insulin, self-application, and storage of insulin. Follow-up lasted six months, with monthly visits. At each visit, all patients received refills of insulin, needles for the pens, and capillary blood glucose strips (three tests/day, at least before breakfast, at lunch, and at dinner, and in the presence of symptoms of hypoglycemia), and they returned the used refills in the subsequent visit to control non-used units. We removed the remaining amount of refills to calculate the number of units of insulin used in the last month. All patients were followed monthly by the same endocrinologist at all visits, conducted in the same tertiary hospital.

In the first month, patients continued to use the same prescribed dose of insulin as before recruitment. After this, adjustments began to be made based on capillary blood glucose annotations according to the protocol or the judgment of the evaluators.

The same endocrinologist (RVM) did adjustments to the therapy in each visit, with the protocol created for this study. Patients did not receive information on the self-adjustment of the doses of insulin. We considered that fasting capillary glucose was appropriate between 70-130 mg/dl. To achieve the target, an adjustment treatment was created adjusting basal insulin (NPH) or rapid acting insulin (regular). To improve the morning control, the evening dose of the NPH insulin was adjusted by increasing or decreasing the previously prescribed dose by 4 IU. To adjust the glycemic control before lunch and dinner, the same change was made in the morning dose of the NPH insulin by 4 IU. For patients using only bedtime NPH insulin who needed to receive another injection in the morning, NPH insulin was introduced at 12 IU before breakfast. In case of a high dose of NPH insulin (greater than 40 IU in each application), regular insulin was adjusted or introduced (4 IU). The regular dose of insulin was adjusted every 4 IU before breakfast, lunch, or dinner (depending on periods without adequate glycemic control).

We asked questions about weekly cases of hypoglycemia and other adverse effects. We reviewed the number of medications, antihypertensive drugs, oral antihyperglycemic agents, and the number of pills taken daily. We measured blood pressure twice on each arm, for the mean value, after relaxing in a sitting position for 10 minutes. We calculated the anthropometric measures of weight, height, and body mass index (BMI) (kg/m^2^).

The questionnaires “Problems Areas in Diabetes - Brazil” (BPAID) and “Diabetes Quality of Life” (DQOL) were applied, both validated in Brazil. The questionnaires used do not have a standardized cut-off point^8^ ^,^ ^9^. The first one evaluates emotional stress related to diabetes in 20 questions. In the second, we used the variables “impact” and “satisfaction” to evaluate quality of life. Other standard variables in this questionnaire are not applicable to the population studied. There were 33 questions. These questionnaires were applied in the first and last visit.

Glycated hemoglobin (ion exchange HPLC) was measured at baseline and 12 and 24 weeks after recruitment. All analyses were done in the same laboratory (*Hospital de Clínicas de Porto Alegre*). We also looked at the medical records to evaluate the measures of HbA1c (same method) at least one year before and six months after the intervention.

The primary endpoint was the reduction of HbA1c in 12 and 24 weeks. Secondary endpoints were the reduction of the number of cases of hypoglycemia or the presence of self-reported nocturnal (need to awaken the patient at night), asymptomatic (only measurement lower than 70 mg/dl without symptoms), or severe hypoglycemia (loss of consciousness or otherwise need to recover). We considered it hypoglycemia when the measured capillary blood glucose was lower than 70 mg/dl or when patients presented symptoms, recovering after eating. We also evaluated reduction of blood pressure levels and improving quality of life. The degree of compliance was also evaluated. Adherent patients were those who used at least eighty percent of the prescribed daily dose in most months, as usually done in clinical studies^4^ ^,^ ^10^ ^,^ ^11^.

To calculate the sample size of 42 patients, we expect an improvement of at least one percent after the use of the insulin pen from baseline. We used a power of 90% and alpha error of 5%. All analyses were performed on an intention-to-treat basis. We applied the Shapiro-Wilk test to evaluate the sample distribution.

Continuous variables with normal distribution were described as mean and standard error (SE) and categorical variables were described as number of cases (percentage). We used the Student's t-test to compare groups with continuous variables. To compare all variables using the same sample, modified in time, categorical variables were analyzed by McNemar's test, and the analysis of continuous variables for repeated measurements was performed by the generalized estimating equation with Bonferroni correction. Analyses were done using the SPSS 18.0 software (Chicago, IL).

## RESULTS

Forty-five patients were included in the study, 35 of whom completed the follow-up. Six patients dropped out because they did not approve the study protocol. One patient had acute myocardial infarction requiring myocardial revascularization surgery and subsequent prolonged hospitalization for surgical site infection. One patient required hospitalization for hip prosthesis infection. One patient suffered lower limb amputation for diabetic foot, and another died without a definite cause during psychiatric hospitalization for alcoholism.

The medical records of the patients or the questions asked on the first visit evaluated their social and demographic characteristics and the medical conditions at baseline, and they are described in Table 1.

**Table 1:** Characteristics of the older patients with uncontrolled TD2 after intervention.

Variable		Measurement
Age in years (mean and SD)		66.71 (4.11)
Male (%)		28.9
Race (%)		
	White	62.2
	Black	24.4
	Other	13.3
Religion (%)		
	Catholic	73.3
	Evangelical	13.3
	Spiritualist	4.4
	Other	8.8
Family Income (%)^a^		
	Up to 1 minimum wage	17.8
	1-2 minimum wages	48.9
	Over 2 minimum wages	33.3
Years of education (%)		
	Uneducated or less than 1 year	8.9
	1 to 3 years	15.6
	4 to 8 years	40.0
	9 years or more	35.6
History of smoking (%)		
	Never smoked	60.0
	Current smoker	-
	Former smoker	40.0
Alcohol consumption (%)		
	Never drank	60.0
	Socially	28.9
	Alcohol abusers	02.0
	Former drinker	09.1
Diabetic retinopathy (%)^b^		
	Absent	11/37 (29.7)
	Mild or moderate non-proliferative	11/37 (29.7)
	Severe non-proliferative	04/37 (10.8)
	Proliferative	11/37 (29.7)
Diabetic nephropathy (%)^c^		
	Absent	17/40 (42.5)
	Increased albuminuria	15/40 (37.5)
	Significantly increased albuminuria	07/40 (17.5)
	Nephrotic-range albuminuria	01/40 (02.5)
Diabetic neuropathy (n and %)^c^		
	Absent	20/39 (51.3)
	Present	19/39 (48.7)
Presence of cerebrovascular disease (n and %)^d^		02/40 (05.0)
Presence of ischemic cardiomyopathy (n and %)^e^		13/43 (31.0)
Time of diabetes in years (mean and SD)		15.93 (7.8)
Time using insulin in years (mean and SD)		9.53 (6.03)
Glycated hemoglobin (mean and SD)		10.34% (0.22)
Positive familiar history for diabetes (%)		64.4
Presence of Hypertension (%)		93.3
Use of sulfonylurea (%)		31.1
Time of hypertension in years (mean and SD)		16.02 (11.09)
Number of medications used (mean and SD)		8.42 (2.32)
Number of antihypertensives (mean and SD)		3.52 (1.08)
Number of pills used (mean and SD)		14.46 (6.42)
Insulin dose per kg/day (mean and SD)		0.85 (0.48)
Regular insulin use (%)		31.1
Body mass index (BMI) (weight/height 2) (mean and SD)		31.70 (4.88)
Systolic blood pressure in mmHg (mean and SD)		138.77 (19.15)
Diastolic blood pressure in mmHg (mean and SD)		70.09 (11.00)

a Minimum wage equal to $220,70 (reference year = August/2015)

b Chart review.

c Chart review; we considered albuminuria > 14 mg/g of creatinine (increased albuminuria) and > 140 mg/g of creatinine (significantly increased albuminuria), and neuropathy in patients with a description of positive monofilament test, sensorial changes, or suggestive lesions.

d History of transient ischemic attack or stroke.

e History of unstable angina, acute myocardial infarction, or diagnosis of ischemic heart disease.

**Figure 1:**
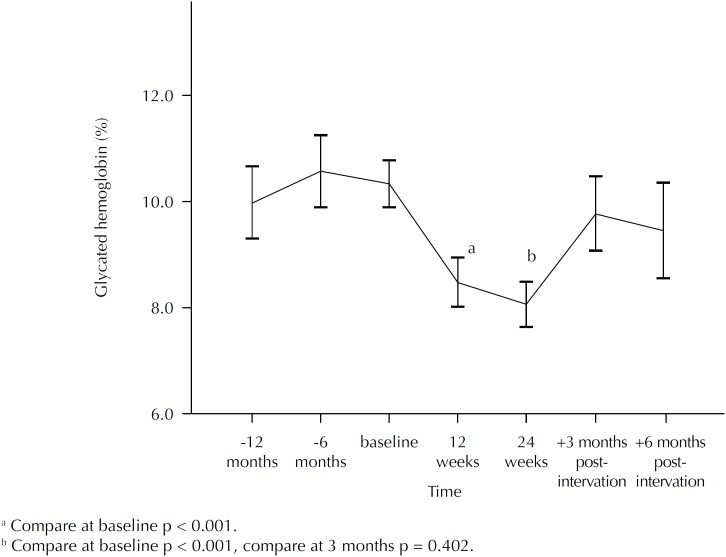
Mean and standard error of glycated hemoglobin before, during, and after the intervention among older patients with uncontrolled TD2.

Mean HbA1c was 10.34% (SE = 0.22) at baseline (range: 8.7% to 14.6%). At 12 weeks of follow-up, HbA1c was 8.54% (SE = 0.24) (p < 0.001). At 24 weeks, HbA1c was 8.09% (SE = 0.21) (difference from baseline p < 0.001). There was no difference between the 12 and 24 weeks (p = 0.402) (Figure 1). There was a reduction in HbA1c of 2.25% during the intervention period. One year before recruitment, HbA1c was 10.08% (SE = 0.32) and 24 weeks before, it was 10.46% (SE = 0.32). Curiously, we monitored the value of HbA1c after the conclusion of study. The results were 9.77% (SE = 0.34) and 9.46% (SE = 0.46) at 12 and 24 weeks respectively, with no difference when compared to baseline values (Figure 1). After completion of the follow-up, patients continued to use the insulin pens and perform blood glucose measurements.

Additionally, we compared patients who used sulfonylurea (14 patients) to those who did not use it (31 patients). The values of HbA1c at baseline were similar in both cases: 10.3% (SE = 1.38%) *versus* 10.4% (SE = 1.59%, p = 0.83). After 24 weeks of follow-up, HbA1c decreased in both groups: −2.42% (SE = 1.49%) *versus* −1.54% (SE = 1.86%, p = 0.43). There was no increased incidence of hypoglycemia between the groups. We found no difference among the prescribed dose of NPH insulin, regular insulin, or the ratio of regular/NPH insulin between users and non-users of sulfonylurea.

Considering the incidence of hypoglycemia per week, we found a frequency lower than once/week (mean 0.8 per week [hypoglycemia reported one month before the inclusion]). These values did not change during the study (p = 1.00). We detected a decrease in the number of patients with asymptomatic hypoglycemia (p = 0.024), but no difference among the number of patients with nocturnal (p = 0.07) or severe hypoglycemia (p = 0.25) during follow-up.

During follow-up, there was an increase in the self-reported average number of medications used from the fourth month onwards, but not in the number of antihypertensive or antihyperglycemic agents, which remained similar during the study. This fact may be explained by other classes of drugs (antidepressants, analgesics, or specific treatment for other comorbidities). Systolic and diastolic blood pressure levels did not change during the study.

**Figure 2:**
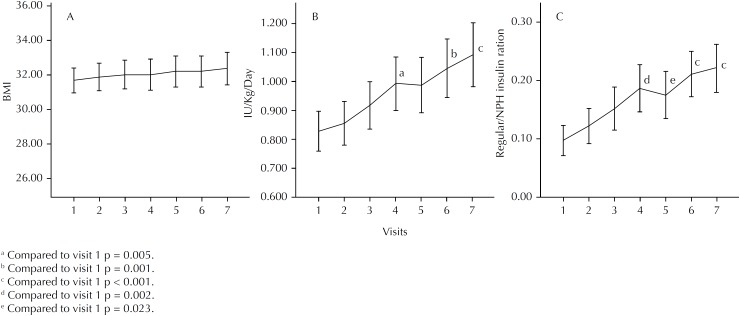
Variation of body mass index and insulin use among older patients with type 2 diabetes without adequate control during follow-up. Mean of the body mass index and standard error (A). Mean of the daily prescribed units of insulin per kilogram of weight and standard error (B). Mean of the daily prescribed units of the ratio of regular to NPH insulin and standard error (C).

In all patients, there was an increase in the prescribed dose of insulin (IU/kg) and in the ratio of regular/NPH insulin from the third month, but the BMI of the patients remained similar to the starting one throughout the study (Figure 2).

**Figure 3:**
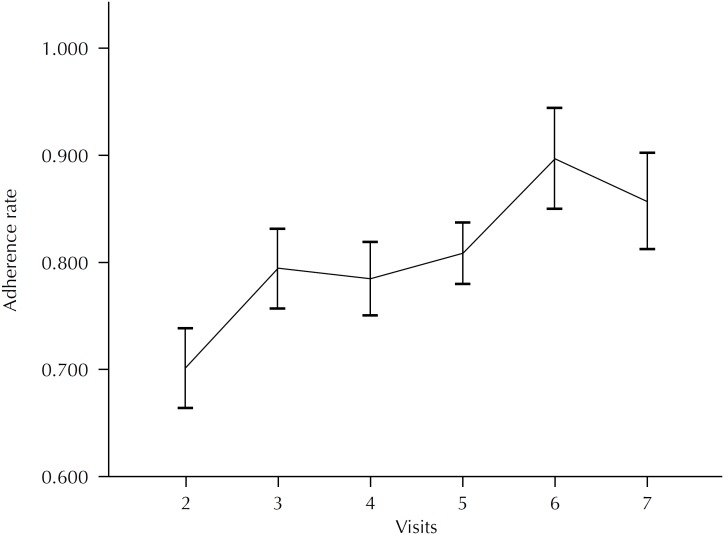
Rates of adherence to insulin use, calculated by the mean of the ratio of used units/prescribed units and standard error.

Regarding adherence to the prescribed dose of insulin, patients used a mean of 70.07% (SE = 3.74) of the prescribed dose of insulin during the first month of study. At subsequent visits, all means were greater than 80% (Figure 3).

Additionally, we evaluated the response to early intervention considering as “responders” patients who reduced HbA1c at least 0.5% in the first trimester of follow-up. Compared to “non-responders”, those patients used more sulfonylureas associated with metformin and insulin (p = 0.001) and had higher rates of hypoglycemia until the sixth visit (p = 0.009), with no differences in severity and presence of nocturnal or asymptomatic hypoglycemia.

We compared pre- and post-intervention quality of life. The scores for BPAID were 39.44 (SE = 3.66) and 34.62 (SE = 4.24, p = 0.107) at baseline and at the end of study, respectively. When we stratified by domains, related to emotions, food, treatment, and social life, we also found no differences. In the DQOL, we used the variables impact and satisfaction. For the variable impact, the scores were 2.27 (SE = 0.11) and 2.43 (SE = 0.12, p = 0.109) at baseline and at the end of study, respectively. For the variable satisfaction, the scores were 2.45 (SE = 0.12) and 2.57 (SE = 0.13, p = 0.109) at baseline and at the end of study, respectively.

## DISCUSSION

In this 24-week study, the multifactorial intervention including the replacement of syringes for insulin pens and the implementation of frequent SMBG, with monthly adjustment in the treatment, was effective to improve glycemic control among older patients with uncontrolled T2D.

In our study, all patients were treated at a public hospital, were already using high doses of insulin, and had chronically uncontrolled T2D despite the efforts of the health care team; therefore, another measure was needed to improve glycemic control. Compared to large clinical trials with T2D^12^ ^-^ ^14^, we found higher levels of HbA1c in the beginning of the study (the Veterans Affairs Diabetes Trial (VADT) had approximately 9.4%, the Action to Control Cardiovascular Risk in Diabetes (ACCORD) trial had 8.1%, and the Action in Diabetes and Vascular Disease: Preterax and Diamicron Modified Release Controlled Evaluation (ADVANCE) trial had 7.2% at baseline). In general, these studies also had the possibility of introducing other oral medication or starting insulin.

A study performed in basic health care^15^ with monthly adjustments in the treatment of diabetes, including SMBG, did not find any improvement in glycemic control in any patient. No improvement in glycemic control was observed even when these authors considered only patients with HbA1c greater than 7% at baseline (mean 8.6% [SE = 1.5%]). The initial mean HbA1c was lower in this group of patients than in our study and only 21.19% of the patients were using insulin. However, many other clinical characteristics were similar and, unlike this study, we provided insulin and pen devices and we asked to conduct more frequent blood glucose tests in our follow-up. Thus, possibly the use of pens may have positively influenced the better results found in our study.

Previous studies have described insulin users as having lower rates of adherence compared to users of oral medications for diabetes treatment^6^ ^,^ ^16^. Adherence to treatment with insulin is usually lower than 75% in patients starting insulin^11^ ^,^ ^16^. However, observational studies including patients who have used insulin for a longer time have shown similar rates of adherence among patients using syringes or pens, both greater than 80%. The long-term use of insulin, regardless of pen or syringe, appears to be related to improved adherence^6^ ^,^ ^10^. Moreover, older patients seem to show better rates of adherence than younger subjects^4^. However, most of these studies are observational and count refills dispensed at the pharmacy, according to medical prescription, to measure adherence^10^ ^,^ ^16^. In our study, the method to evaluate adherence appears to be more precise than in other studies. We counted the amount of units of insulin in each refill. Nonetheless, we found higher rates of adherence to treatment as described in the literature from the second month of follow-up.

Another factor that may have justified a significant improvement in glycemic control was the possibility of frequent adjustments of insulin. In general, before recruitment, patients were seen every 4-6 months, which is a long period between evaluations. During follow-up, we adjusted the prescribed dose of insulin every month based on the capillary blood glucose. When this intervention was suspended, even after keeping patients on the insulin pen, the level of HbA1c returned to values similar to baseline. Although the inclusion criterion was the most recent value of HbA1c, all patients had HbA1c similar to this value (more than 8.5%) at least 24 weeks before recruitment (with chronically poor glycemic control).

The Self-monitoring of Blood Glucose may also have contributed to the improvement of glycemic control, but not all results can be justified by this management alone. Results presented in previous studies are inconsistent given the variety of methodologies and different frequencies of capillary glucose measurements^17^ ^-^ ^20^. Our patients underwent three measurements daily. A systematic review with meta-analysis has shown a slight decrease in favor of SMBG, however with multiples protocols of capillary glucose^21^. In addition, the direct supply of insulin pens, insulin refills, and lancets may have benefited the glycemic control. Patients received inputs for the following month. Facilitating access to treatment may have influenced outcomes.

Usually, insulin is the third drug included in T2D treatment in the public health system in Brazil, with free distribution, after attempting treatment with metformin and sulfonylurea. A systematic review and network meta-analysis has assessed the potential decrease of HbA1c with various classes of drugs as a third drug. Although the results were similar between oral drugs and insulin, the original studies have used much lower doses of insulin than what is usually necessary for patients with very poor glycemic control, as in our study. Perhaps the effect of insulin has been underestimated^22^. Current evidence reinforces the maintenance of sulfonylurea treatment in patients with T2D when there is secondary failure with this class of drugs, with compulsory association with insulin^23^ ^-^ ^25^. However, we received some patients who were not yet using this drug, and who were using high doses of insulin. In this situation, we did not add sulfonylurea in order to not alter the study protocol. Additionally, we did no suspend sulfonylurea, only the adjustment of the dose of insulin. We found no difference between patients with or without sulfonylurea. However, we believe that, regardless of the prescribed dose of insulin, the maintenance of sulfonylurea may help in the glycemic control.

A possible limitation was the absence of a control group to evaluate the effect of using insulin pens (non-randomized study). On the other hand, our objective was to evaluate a multifactorial strategy to improve glycemic control in chronically decompensated patients treated in the Brazilian Unified Health System. One year before recruitment, with conventional medical care, patients did not have satisfactory glycemic control. We also realized that patients found it extremely difficult to understand the questionnaires; even with poor glycemic control at baseline, they said that they were very satisfied with the result of their treatments, clearly showing the poor understanding of their glycemic control and disease. Although several of them said that they preferred to use pens, we could not measure this information with validated instruments. One limitation was the small number of participants representing a large group of patients with T2D. We also had a high rate of drop-outs; despite that, the expressive result in the decrease in HbA1c was statistically significant and the intention-to-treat analysis can minimize this limitation.

We found no difference regarding hypoglycemia events. However, the mean HbA1c level was higher in the beginning of the study and even lower than baseline; the final values were well above the target. Therefore, if more patients had reached the HbA1c levels near 7%, we would possibly have more hypoglycemic events per patient. During the ACCORD trial^13^, the group achieved 6.4% of HbA1c on average, compared with the group that achieved 7.5% of HbA1c on average; therefore, they had higher rates of hypoglycemia and, consequently, needed medical care. In addition, very low levels of HbA1c among high-risk patients result in increased mortality, but the rationale for this fact remains unexplained^26^.

In conclusion, strategies to improve the glycemic control in older subjects with T2D work, but only during the period of the study with all interventions implemented at the same time. The offer of more frequent medical visits and devices to patients can contribute with these strategies. The individual effect of each strategy still needs to be further studied.
